# Identification of Magnesium Oxychloride Cement Biomaterial Heterogeneity using Raman Chemical Mapping and NIR Hyperspectral Chemical Imaging

**DOI:** 10.1038/s41598-018-31379-5

**Published:** 2018-08-29

**Authors:** Ronan M. Dorrepaal, Aoife A. Gowen

**Affiliations:** UCD School of Biosystems and Food Engineering, University College Dublin, Dublin, Ireland

## Abstract

The present study investigated spatial heterogeneity in magnesium oxychloride cements within a model of a mould using hyperspectral chemical imaging (HCI). The ability to inspect cements within a mould allows for the assessment of material formation in real time in addition to factors affecting ultimate material formation. Both macro scale NIR HCI and micro scale pixel-wise Raman chemical mapping were employed to characterise the same specimens. NIR imaging is rapid, however spectra are often convoluted through the overlapping of overtone peaks, which can make interpretation difficult. Raman spectra are more easily interpretable, however Raman imaging can suffer from slower acquisition times, particularly when the signal to noise ratio is relatively poor and the spatial resolution is high. To overcome the limitations of both, Raman/NIR data fusion techniques were explored and implemented. Spectra collected using both modalities were co-registered and intra and inter-modality peak correlations were investigated while k-means cluster patterns were compared. In addition, partial least squares regression models, built using NIR spectra, predicted chemical-identifying Raman peaks with an R^2^ of up to >0.98. As macro scale imaging presented greater data collection speeds, chemical prediction maps were built using NIR HCIs.

## Introduction

Magnesium oxychloride (MOC) cements were first reported by Sorel in 1866^1^. Several MOC cement crystal phases exist, however only two phases form below 100 °C. These are phases 3 (P3) and 5 (P5)^2^, so named according to the relative molar ratios of the MgO and MgCl_2_ constituent components (P3: 3Mg(OH)_2_·MgCl_2_·8H_2_O and P5: 5Mg(OH)_2_·MgCl_2_·8H_2_O)^3^.

The initial popularity of MOC cements stemmed from their rheological properties, which proved to be superior to Portland cement, complemented by their rapid setting and high early strength characteristics^4^. These properties enable quick repair as well as the ability to flow into irregular cavities. However, MOC cements never reached widespread use due to their relative instability in water, degrading to lower strength brucite^4^. More recently, modifying additives, in particular phosphate species, have been suggested as a means of improving the water resistance of MOC cements, and preventing cement degradation to brucite^4,5^. Studies^6–10^ have also shown magnesium phosphate based cements to potentially be a strong alternative to calcium phosphate cements in certain applications, as they may achieve a more ideal combination of mechanical strength, more rapid setting time and preferred resorption rate than calcium phosphate cements, while remaining biocompatible *in vitro*^11^. Inherent antibiotic behaviour has also been reported in relation to magnesium phosphate cements^11–13^. These factors have opened up the study of MOC cements and their phosphate derivatives for biomaterial applications^4,14^.

Generally, in the study of MOC cements, analytical measurements are taken in relation to bulk properties which are assumed to apply to the material as a whole. In particular, Fourier Transform Infra-Red (FTIR) spectroscopy^4,15,16^, Raman spectroscopy^16^ and X-Ray Diffraction (XRD)^3–5^ results have been reported. Typically, spectra recorded from these techniques are acquired from a small number of sample points and are averaged in order to arrive at a representation of the studied material.

However, more recently, efforts have been made to move beyond traditional point spectral acquisition, towards a spatial-spectroscopic technique known as hyperspectral chemical imaging (HCI)^7,17^. For example, Bannerman *et al*.^7^ demonstrate the Raman chemical mapping of brushite (CaHPO_4_.2H_2_O) based calcium phosphate ceramics. In that study, Raman chemical mapping was used revealing spatial heterogeneity in the cement ageing process. Moreover, this study demonstrated that point spectroscopy is unlikely to provide adequate information of the local ageing of a cement specimen.

In another study, Koburger *et al*.^18^ demonstrated a method for the monitoring of mineralisation in hydrogels at the engineered hard-soft tissue interface. A brushite cement/hydrogel interface was investigated for calcium phosphate deposits which suggested ion migration.

The present study investigated chemical spatial heterogeneity of cement surfaces in a non-destructive manner. This was achieved through the use of a novel experimental arrangement designed to model a mould, where cement specimens were allowed to set and be imaged between glass slides. The arrangement draws parallels with previous studies^19,20^, where polymer matrix pharmaceutical tablets, trapping active ingredients, were confined within flow cells and drug solubilities were monitored. ATR-FTIR imaging in combination with Raman mapping^19^, and NIR imaging in combination with Raman mapping^20^ were performed allowing for the detection of *in situ* dissolution of pharmaceutical active ingredients. These studies differed from the present study in that different specimens were used between spectral modalities. While an insight can be gained in relation to the dissolution mechanism generally, direct comparisons between imaging modalities cannot be assessed where different specimens are investigated. Based on the information given, it appeared necessary that non-contiguous Raman maps rather than contiguous Raman images were acquired in the above studies^7,18–20^. Due to the time constraints involved in collecting complete Raman images with micron scale pixels over macroscopic scale samples, it is typical to obtain Raman images from small sub-regions of a specimen or to sample larger regions by mapping, where the step size is considerably larger than the pixel size (chemical mapping is discussed in greater detail in Dorrepaal and Lawless *et al*.^21^).

## The aim of the present work was to study spatial heterogeneity in MOC cements using HCI by the fusion of NIR and Raman data for more extensive surface characterisation

Both NIR HCI and Raman chemical mapping were employed to characterise the same cement specimens. Raman mapping was considered to be the primary acquisition technique, as the Raman spectra of MOC cements are well characterised^16^ with distinctive and characterisable chemical peaks. In the present study, NIR imaging benefitted from far faster acquisition times than Raman data acquisition, where equivalent specimen areas were investigated, allowing the potential for rapid evaluation of large numbers of specimens. However NIR imaging appears to be greatly under-utilised in the chemical characterisation of MOC cements. To the authors’ knowledge, just one study of MOC cements employed the NIR imaging^17^, though the focus of that study was not chemical characterisation. NIR imaging may have been limited previously due to difficulties incurred from spectral convolution through the overlapping of signal overtones, which can make interpretation difficult. In the present study, data fusion techniques (such as those discussed in^22–24^) were used to investigate the viability of NIR imaging in the assessment of spatial heterogeneity of MOC cement crystal phases, through the ability of NIR spectra to predict distinguishable chemical features observed in Raman spectra.

## Results and Discussion

### Spectral characteristics and correlation

#### Raman spectra intra-correlation

**Figure 1:**
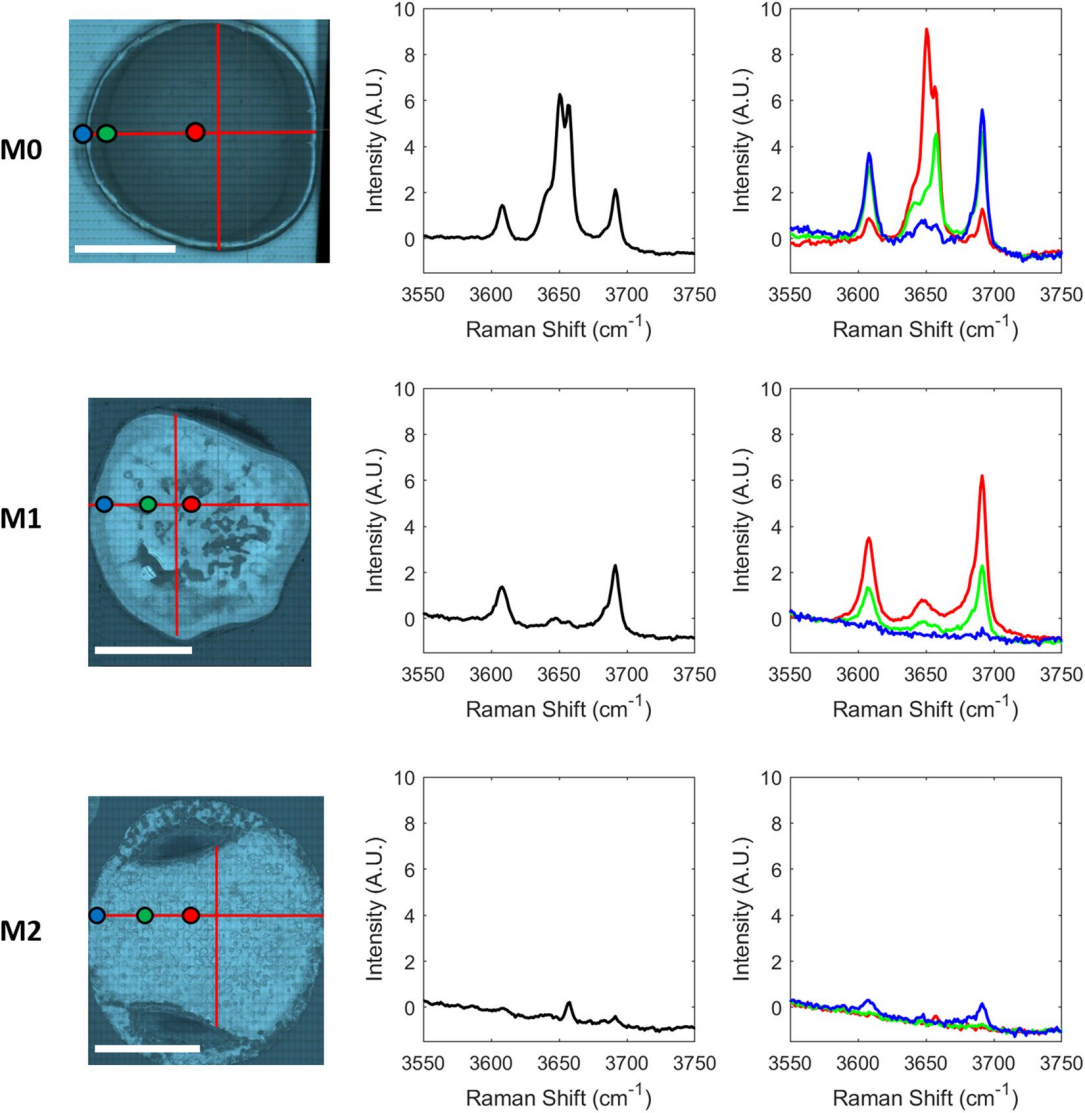
3550 cm^−1^ to 3750 cm^−1^ region of mean M0, M1 and M2 Raman map spectra (middle column) and spatially distributed M0, M1 and M2 point spectra (right column) with colour coded reference to position of composite white light image (left column; red lines indicate regions of Raman spectral acquisition; white scale bar length = 1 cm).

For each map, the mean spectrum was calculated. However, upon pixel-wise inspection of map spectra, it was found that the calculated mean spectra were not representative of specimens as a whole. Instead, the cements proved to be chemically heterogeneous in nature, differing with distance from the boundary region (Fig. 1).

Five sharp peaks are present in the M0 Raman spectra (Fig. 1, top-right), found at 3608 cm^−1^, 3639 cm^−1^, 3650 cm^−1^, 3657 cm^−1^ and 3691 cm^−1^. In order to assess which peaks tend to occur in tandem, Pearson correlation analysis was conducted on the M0 cement spectra (Supplementary Table 1). A high (~0.98) correlation coefficient exists between the peaks found at 3608 cm^−1^ and 3691 cm^−1^, as well as between the peaks found at 3639 cm^−1^ and 3657 cm^−1^ (~0.95). High correlations such as these are generally indicative of peaks which describe aspects of the same chemical feature or crystal structure. The peaks are identified as the OH stretching vibrations of P5 MOC cement (3608 cm^−1^ and 3691 cm^−1^) and the OH stretching vibrations of P3 MOC cement (3639 cm^−1^ and 3657 cm^−1^), as reported by Kanesaki and Aoyama^16^. While there is a small linear offset in the positioning of the P3 and P5 peaks relative to that study, it is believed that this is most likely due to the use of different instrumentation calibration protocols. Further, P5 peaks are also found at 3608 cm^−1^ and approximately 3691 cm^−1^ of the IR spectra presented by Tan *et al*.^4^. The Raman peak at 3650 cm^−1^ showed no large positive correlation with any other measured Raman shifts and is identified by Duffy *et al*.^25^ as an OH stretching vibration of brucite. Peak characterisation information is presented in Table 1.

**Table 1:** MOC Raman peak characterisation^4,16,25^.

Species	Raman Shift
P5 MOC Cement	3608 cm^−1^ (weak), 3650 cm^−1^ (very weak) and 3691 cm^−1^ (weak)
P3 MOC Cement	3639 cm^−1^ (medium) and 3657 cm^−1^ (strong)
Brucite	3650 cm^−1^ (very strong)

Looking at the Raman M1 map spectra (Fig. 1, middle row, right column), two strong peaks are visible at 3608 cm^−1^ and 3691 cm^−1^. These peaks show a very high Pearson correlation value of ~0.99 and are again identified as P5 MOC cement^16^. Interestingly, the feature at 3650 cm^−1^ shows high Pearson correlations of ~0.96 and ~0.95 with 3608 cm^−1^ and 3691 cm^−1^, respectively. This was not the case for the M0 sample and can be explained by the fact that a weak peak attributed to P5 MOC cement appears at this position but can be convoluted by a brucite peak at the same position. In the absence of brucite, a strong correlation is calculated between the peaks at these Raman shifts. Likewise, in the absence of P3 MOC cement, there is a relatively strong correlation (Pearson correlation coefficient ranging from 0.77–0.91) between measurements at 3639 cm^−1^ or 3657 cm^−1^ with all other peaks of interest, due to the neighbouring P5 peak which centres at about 3650 cm^−1^, and whose signal extends partially over those measured Raman shifts. Visual inspection of the spectra confirmed the presence of P5 MOC cement. The majority of M2 Raman spectra did not comprise any of the strong OH peaks of interest. Supplementary Table 1 presents Pearson correlation data relating to the M2 Raman spectra. The peaks at 3639 cm^−1^ and 3657 cm^−1^ again correlate very strongly (0.95) with one another and, upon inspection, P3 MOC cement peaks were found in a small number of M2 Raman spectra. There is also a relatively strong correlation value (0.87) found between the peaks at 3608 cm^−1^ and 3691 cm^−1^ and again small numbers of P5 MOC cement peaks were found. From visual inspection of the spectra, it does not appear that a brucite peak is present at 3650 cm^−1^.

The Pearson correlation coefficients were also calculated for the entire Raman dataset regardless of cement type (Supplementary Table 1). Looking at the five OH peaks of interest, the strongest correlation is found between the P5 peaks at 3608 cm^−1^ and 3691 cm^−1^ with a Pearson coefficient of ~0.99. Interestingly, these peaks do not correlate with the measurement at 3650 cm^−1^. A strong correlation is also found between the P3 peaks at 3639 cm^−1^ and 3657 cm^−1^ with a Pearson coefficient of ~0.98. Finally relatively strong Pearson coefficients are found between 3650 cm^−1^ and both 3639 cm^−1^ and 3657 cm^−1^. As stated, this is likely due to the spreading of correlated P3 peaks centred at 3639 cm^−1^ and 3657 cm^−1^ over the measurement at 3650 cm^−1^, in the absence of brucite.

#### NIR spectra intra-correlation

**Figure 2:**
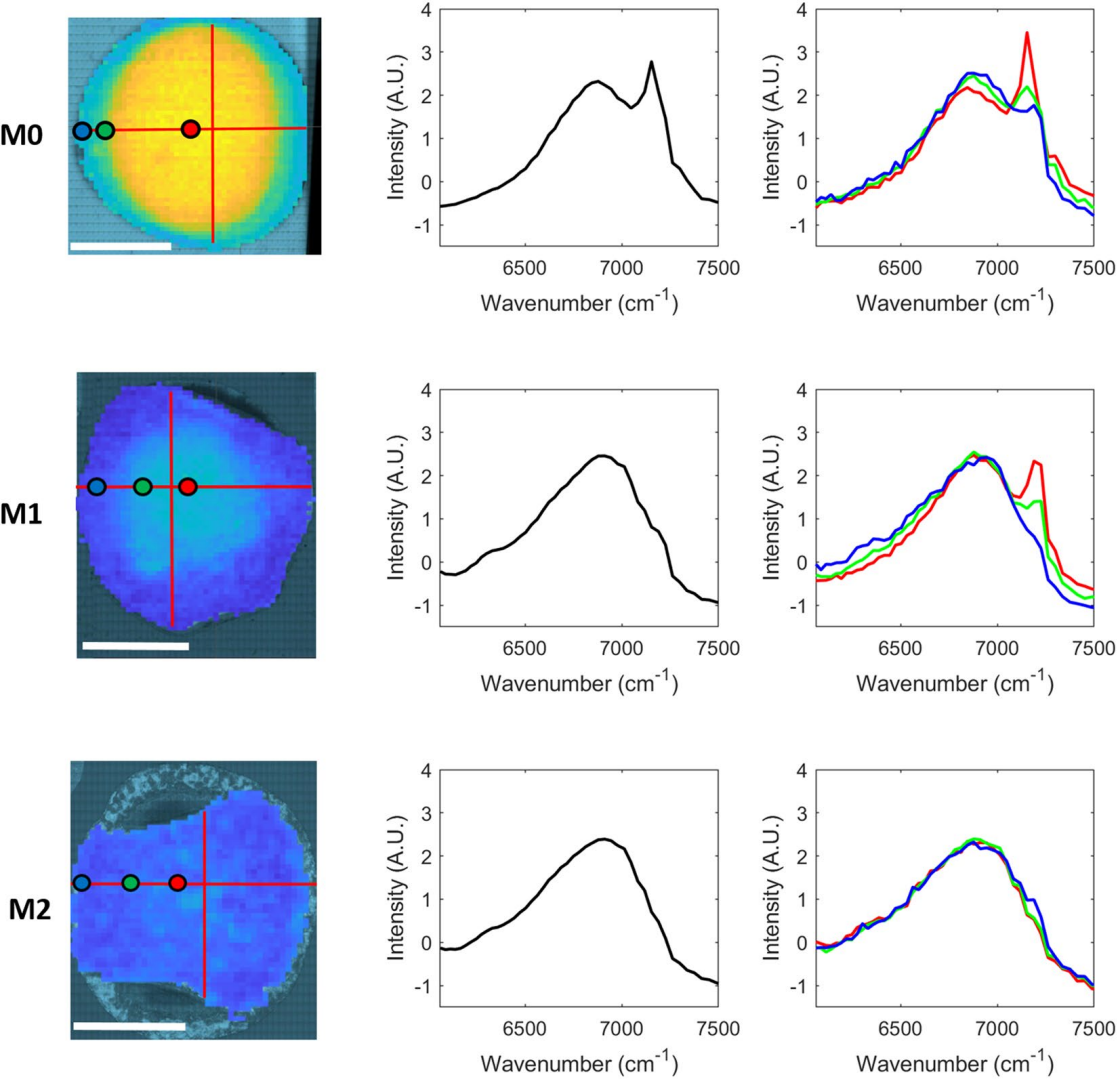
6060 cm^−1^ to 7500 cm^−1^ region of mean M0, M1 and M2 NIR image spectra (middle column) and spatially distributed M0, M1 and M2 point spectra (right column) with colour coded reference to NIR image rendered at 7153 cm^−1^ (left column; red lines indicate regions for co-registration with Raman spectra; white scale bar length = 1 cm).

NIR images were acquired across the entire surface of the M0, M1 and M2 cement specimens and spectral correlation analysis was performed. A broad water band can be seen in all mean spectra (Fig. 2; middle column), however a strong peak is also present at 7153 cm^−1^ in the case of the M0 spectrum. It appears that a shoulder may be emerging at a similar position in the mean M1 spectrum but no equivalent peak is visible in the mean M2 spectrum. Fig. 2 (right column) presents spatially distributed M0, M1 and M2 NIR point spectra.

Supplementary Table 2 presents the Pearson correlation coefficients between selected NIR wavenumbers of the broad OH band. In the case of the M0 specimen, the strongest correlations were found between 7336 cm^−1^ and 7153 cm^−1^ as well as between 7189 cm^−1^ and 7153 cm^−1^, with correlation coefficients of ~0.94 and ~0.92 respectively. This differed from the M1 specimen which showed high correlations (≥0.96) between all combinations of 7336 cm^−1^, 7225 cm^−1^, 7189 cm^−1^ and 7153 cm^−1^. The M2 specimen showed the strongest correlation between 7225 cm^−1^ and 7189 cm^−1^ with a correlation coefficient of 0.95. When the Pearson correlation was calculated on the NIR spectra of all cements, the greatest values were found between 7336 cm^−1^ and 7189 cm^−1^ as well as 7153 cm^−1^ (0.91 and 0.94 respectively), 7225 cm^−1^ and 7189 cm^−1^ (~0.97), and 7189 cm^−1^ and 7153 cm^−1^ (~0.93).

#### Inter-modality correlation

**Figure 3:**
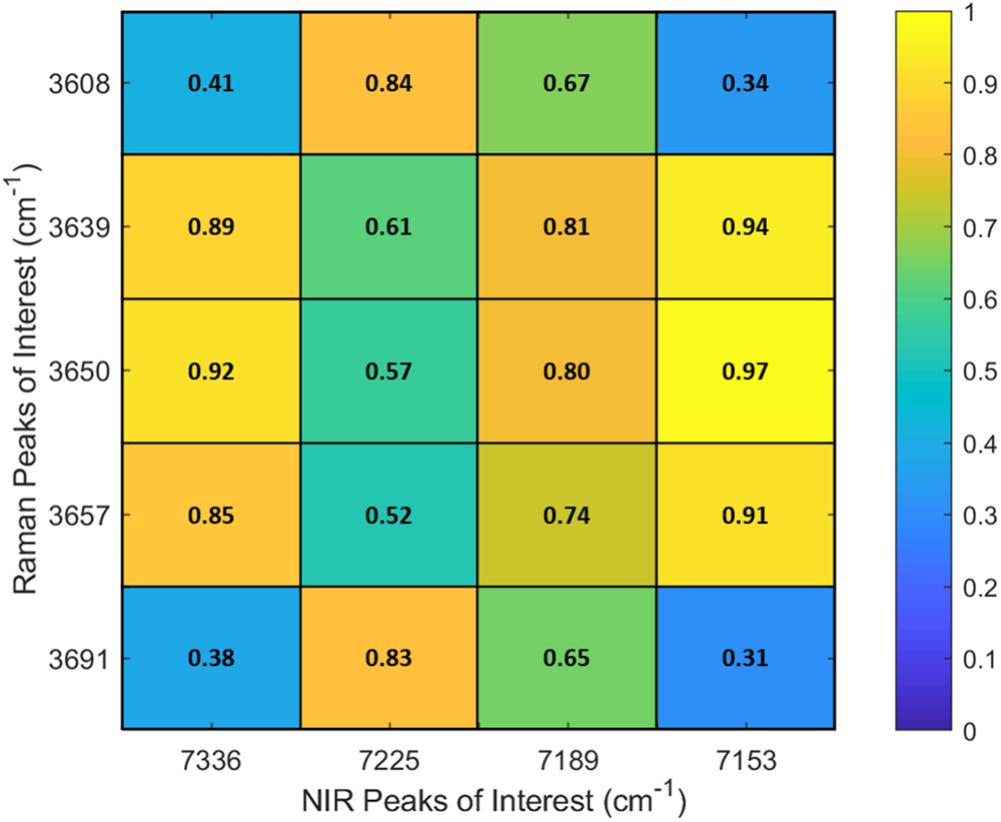
Pearson correlation values between NIR peaks of interest in M0, M1 and M2 MOC with Raman peaks of interest.

NIR features generally proved to be broad, making characterisation from NIR spectra alone difficult. Inter-modality Pearson correlations between Raman and NIR spectra were therefore investigated (peaks of interest: Fig. 3, full spectral range: Supplementary Fig. 1). It can be seen that strong correlations are generally found between the Raman brucite peak at 3650 cm^−1^ with 7336 cm^−1^ and 7153 cm^−1^ of the NIR range (~0.92 and ~0.97 respectively). The same trend appears between the Raman P3 peaks at 3639 cm^−1^ and 3657 cm^−1^ and the NIR measurements at 7336 cm^−1^ and 7153 cm^−1^ (though with slightly lower correlation values). These correlations suggest that the NIR peaks at 7336 cm^−1^ and 7153 cm^−1^ might be indicative the presence of brucite, P3 or both. Some correlation between the P5 Raman peaks at 3608 cm^−1^ and 3691 cm^−1^ and the NIR measurements at 7189 cm^−1^ and 7225 cm^−1^ is also seen. While the correlation values of ~0.84 and ~0.83 are not particularly high, it is noteworthy that these similar values occur for both Raman P5 peaks in tandem and are considerably higher than correlation values for the NIR 7225 cm^−1^ peak with every other Raman peak of interest. As previously stated, NIR peaks are broad, and combined with the relatively low spectral resolution of 7 nm, it is possible that this limitation has affected the correlation result. In addition, univariate comparisons may not be sufficient for comparison between modalities. For this reason, multivariate approaches were investigated.

### Unsupervised clustering: k-means

In order to assess the spatial arrangement of chemical entities across the cement surfaces, k-means clustering was performed on all Raman spectra. K-means clustering was also performed on the portions of the NIR images which co-locate to the Raman maps. The number of clusters was selected using the elbow method averaged over 10,000 iterations, where 6 clusters were selected for Raman data and 5 clusters were selected for NIR data (Supplementary Figs 2, 3).

**Figure 4:**
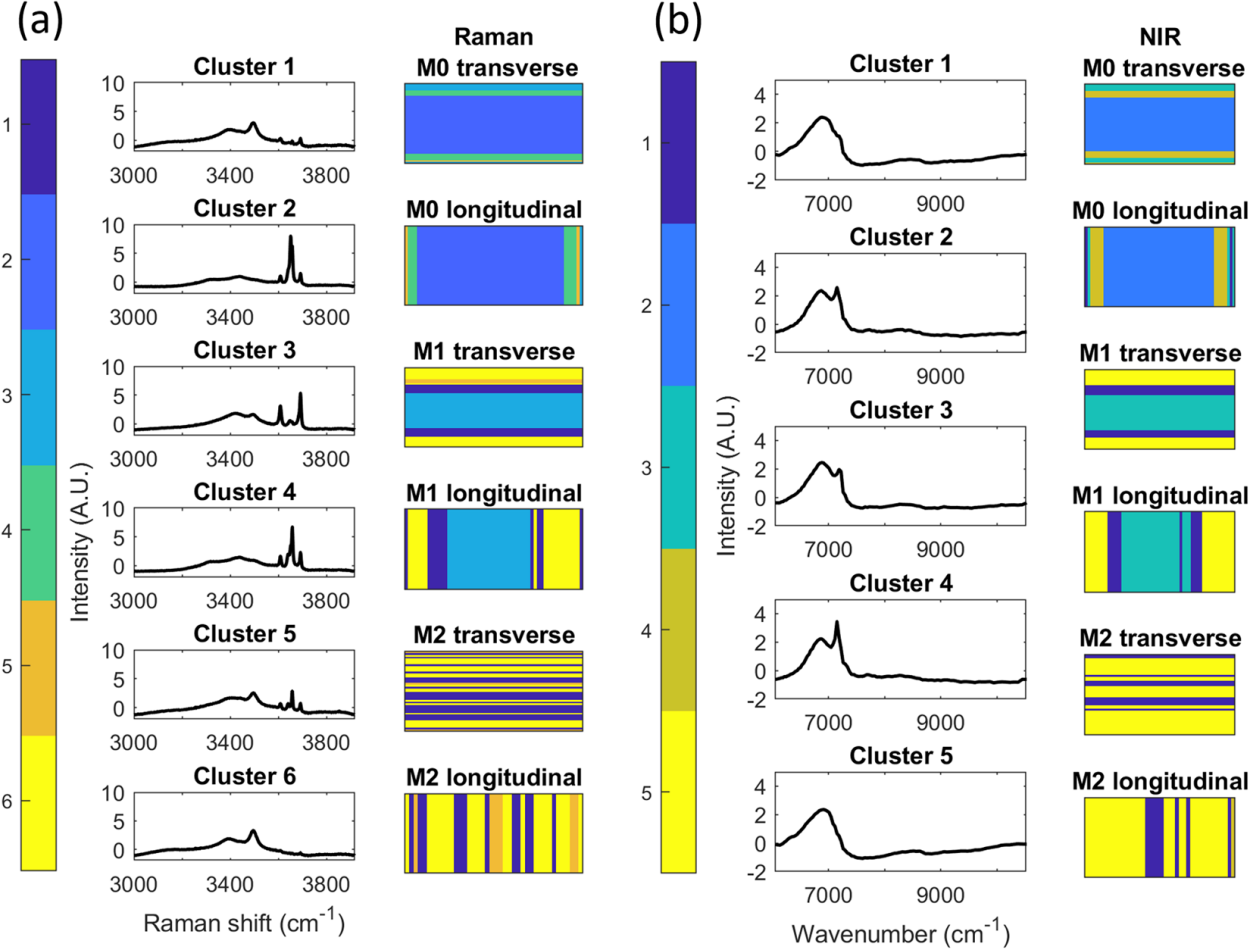
**(a)** Raman k-means cluster (k = 6) images M0, M1 and M2 in longitudinal and transverse directions with cluster centre. **(b)** NIR k-means cluster (k = 5) images M0, M1 and M2 in longitudinal and transverse directions with cluster centre.

The k-means cluster assigned to each pixel is presented spatially in Fig. 4 [(a) Raman, (b) NIR] in addition to the related cluster centroids. Raman cluster identification, based on visual inspection of the centroid spectra, is presented in Table 2.

**Table 2:** Chemical identification of Raman k-means clusters as presented in Fig. 4 (based on literature^4,16^).

Raman Cluster	Raman Cluster Centroid Identification
1	P3 [very weak peaks] + P5 [very weak peaks]
2	Brucite, P3 and P5
3	P5
4	Brucite [weak], P3 and P5
5	P3 [weak peaks] + P5 [very weak peaks]
6	P5 [very weak peaks]

Raman clusters 2 and 4 represents regions of brucite, P3 and P5, where cluster 2 has a larger brucite peak. Clusters 1 and 5 represent regions of P3 and P5, where the peaks of cluster 5 are larger than those of cluster 1. Cluster 3 represents regions of P5 only, with small P5 peaks visible in cluster 6. The broad, overlapping OH and H_2_O bands in the 3100 cm^−1^ to 3550 cm^−1^ range varied between clusters. As can be seen in Fig. 4(a), there is a classification trend based on the relative position of acquired Raman spectra for both the M0 and M1 specimens (in both longitudinal and transverse orientations), central pixels are differently classified to those near the edges.

It can be seen that the general spatial trend is similar for both the Raman and NIR clustering (Fig. 4). The greatest divergence in k-means results was between Raman and NIR clustering of M2 cement, where the Raman data clustered to clusters 1 and 6 (NIR clusters 1 and 5 were generally found in similar regions of M1 and M2) in addition to Raman cluster 5 which did not have an equivalent in NIR clustering (clusters were selected using the elbow method, and one fewer cluster was used in the case of NIR). The difference may be due to a lack of modality sensitivity (Raman or NIR’s inability to differentiate chemical species), however it may be due to the difference in pixel acquisition area between modalities, where the Raman acquisition area is considerably smaller than that of NIR. It may be that cluster 5 components found in Raman data is present in smaller areas than the NIR pixel size.

**Figure 5:**
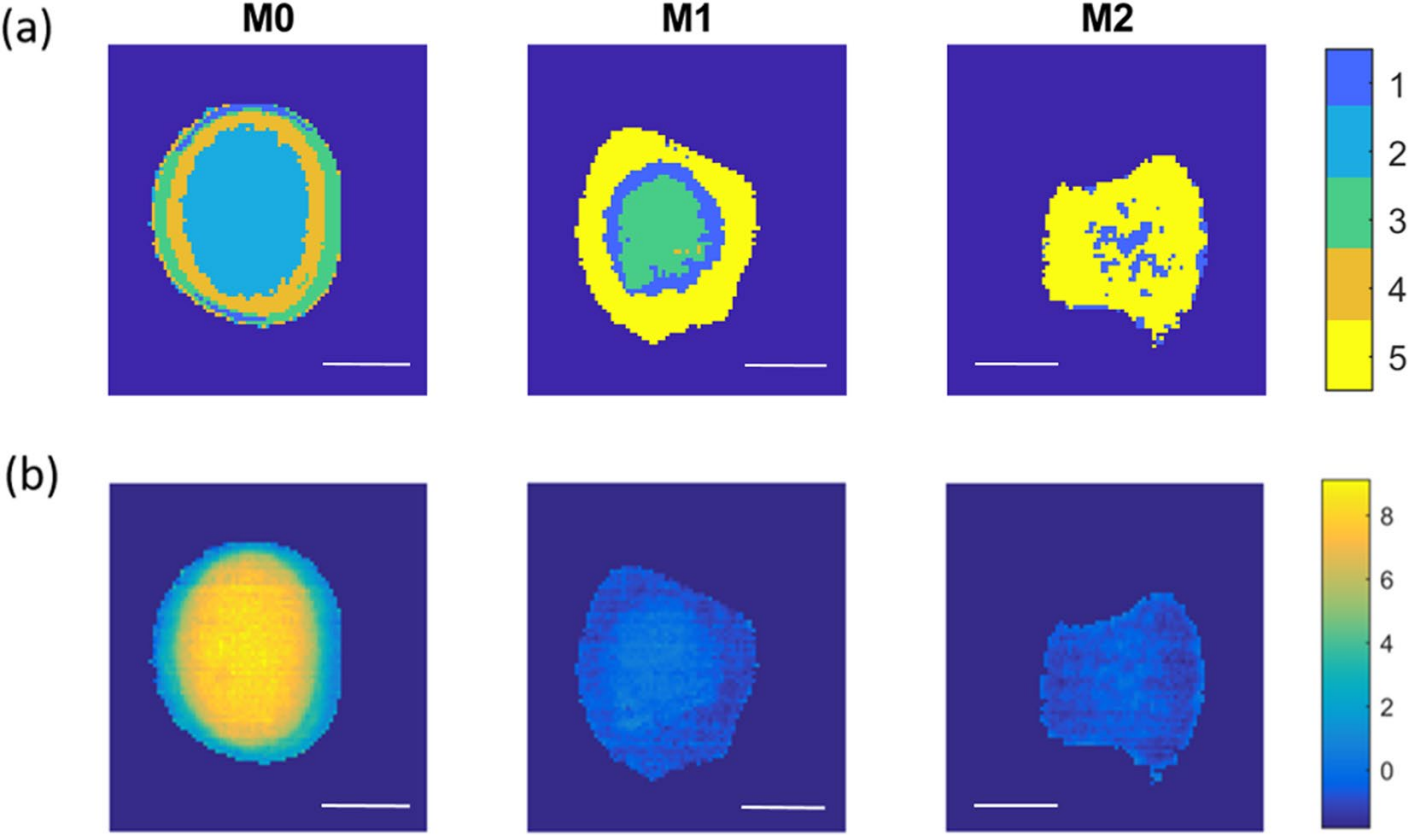
**(a)** MOC cement k-means cluster images generated from minimum Euclidean distance calculations relative to NIR cluster centres of Fig. 4(b). **(b)** PLS prediction of 3650 cm^−1^ Raman shift images from NIR HCIs. White scale bar length = 1 cm.

As unsupervised clustering appears to have performed well (particularly in the case of M0 and M1), the Euclidean distance between all NIR image spectra and each cluster centroid was calculated. Each pixel was then identified as the cluster with the closest cluster centroid. The resulting cluster maps are presented in Fig. 5(a) and demonstrate the differences in spatial distribution of components both within and between samples. It can be seen that the M0 specimen (Fig. 5(a), left) was separated into four distinct regions; a central region (cluster 2: brucite, P3 and P5), an inner boundary region (cluster 4: brucite [weak peak], P3 and P5), an outer boundary region (cluster 3: P5) and two small regions at the top and bottom of the image (cluster 1: P3 [very weak peaks] + P5 [very weak peaks]). A trend is clearly visible when comparing the centre to the boundary regions with similar trends evident in the cases of the M1 and M2 specimens. M1 is separated into three separate regions; a central region (cluster 3: P5), an inner boundary region (cluster 1: weak P5) and an outer region (cluster 5: P5 [very weak peaks]). M2 is separated into two regions, one central region (cluster 1: weak P5) and a surrounding region (cluster 5: P5 [very weak peaks]).

### Partial Least Squares prediction of Raman spectral features from NIR spectra

As NIR image acquisition time was orders of magnitude faster than Raman acquisition of an equivalent area, the acquired NIR spectra were tested for their ability to predict spectral features identifiable by Raman spectroscopy. Of the Raman shifts of interest, it was found that prediction of the 3650 cm^−1^, generally indicative of brucite, showed the greatest coefficient of determination of prediction (R^2^_p_) value (Table 3), a result in agreement with k-means results. Brucite is a weak, generally undesirable product which can form in the synthesis of MOC cements or over time due to MOC cement degradation in water^4^ and therefore, its identification using NIR is desirable.

**Table 3:** PLS model results for Raman shifts of interest, where R^2^_p_ is the coefficient of determination of prediction and nLV is the number of latent variables used in the PLS prediction model.

Raman Shift (cm^−1^)	3608	3639	3650	3657	3691
R^2^_p_	0.7745	0.8898	0.9843	0.8746	0.8117
nLV	4	4	4	4	4

The PLS model generated was applied to the full NIR images in order to predict Raman intensities at 3650 cm^−1^ for each NIR pixel (Fig. 5(b)). High intensity values were only predicted in the central region of the M0 cement, with low predicted values for all pixels of both the M1 and M2 cements.

The PLS regression vector was found to be highly interpretable, as it can be seen to select for the 3650 cm^−1^ Raman shift by the positive weighting of the NIR measurement at 7153 cm^−1^ and the negative weighting of the NIR measurement at 7225 cm^−1^ (Supplementary Fig. 4). Therefore the regression vector not only provided an accurate model for prediction, but also provided an insight into the interpretation of the NIR MOC spectra using the Raman MOC spectra.

The study presents a model for the assessment of materials as they might exist within a mould and demonstrates the risk in any assumption of specimen homogeneity during the setting process. The consequences of these findings are significant. Conventional characterisation of biomaterials is carried out on a bulk basis, usually involving one average measurement per sample. For example, the assessment of cement materials by the pulverisation of cements followed by the assessment of a small portion of the resultant powder for inspection of material chemical/crystal structure^4,18,26^. However, such bulk analysis can lead to sampling bias through the assessment of only localised cement, or alternatively, the averaging of data collected from heterogeneous cement. Further, the destructive force applied during pulverisation, as well as the resultant increased cement surface area, may affect specimens and subsequent measurements. The developed approach enabled prediction of the brucite Raman peak on macroscale samples using NIR imaging data. This approach could be applied in online testing, for example in manufacturing environments or on-site inspection of cements using hyperspectral cameras operating in the NIR wavelength range.

## Conclusion

The presented experimental arrangement can be used as a model for the analysis of biomaterials as they form within a mould. It has been demonstrated that the chemical/crystal structure of a material can be manipulated by its environment during formation. Further, it has been shown that such differences can be detected *in situ* during setting using HCI. HCI can be used to grant insight into material formation during setting or after setting. Finally, it has been shown that while precise chemical identification can be performed using Raman spectroscopy in many cases, multivariate models can be built for use with NIR, with the potential for high accuracy in the prediction of classifying Raman chemical peaks. These models may prove useful in instances where NIR imaging is faster and can cover greater surface areas. As such there is a potential for NIR imaging in the assessment of the quality of large volumes of biomaterial cements in addition to, or replacement of, Raman spectroscopy where large scale contiguous HCI data acquisition has proved burdensome.

## Materials and Methods

### Cement production

An MOC cement and two phosphoric acid MOC derivative cements were prepared. The sample types were named M0, M1 and M2 based on the phosphoric acid content of the aqueous solution used during synthesis (0 molar, 1 molar and 2 molar respectively).

#### M0 synthesis

MgCl_2_.6H_2_O (Applichem GmbH, Ottoweg, Damstadt, Germany; 2.4398 g) was dissolved in DI water (3 mL). MgO (Fisher Scientific UK, Bishop Meadow Road, Loughborough, UK; 3 g) was then added to the solution and the mixture was stirred vigorously until a paste formed.

#### M1 and M2 synthesis

MgCl_2_.6H_2_O (3.6595 g) was dissolved in phosphoric acid solution (1 M and 2 M diluted from ≥85% phosphoric acid, Sigma Life Science, Steinheim, Germany; 4.5 mL). When dissolved, MgO (3 g) was added and stirred vigorously until a paste formed.

### Cement setting protocol

**Figure 6:**
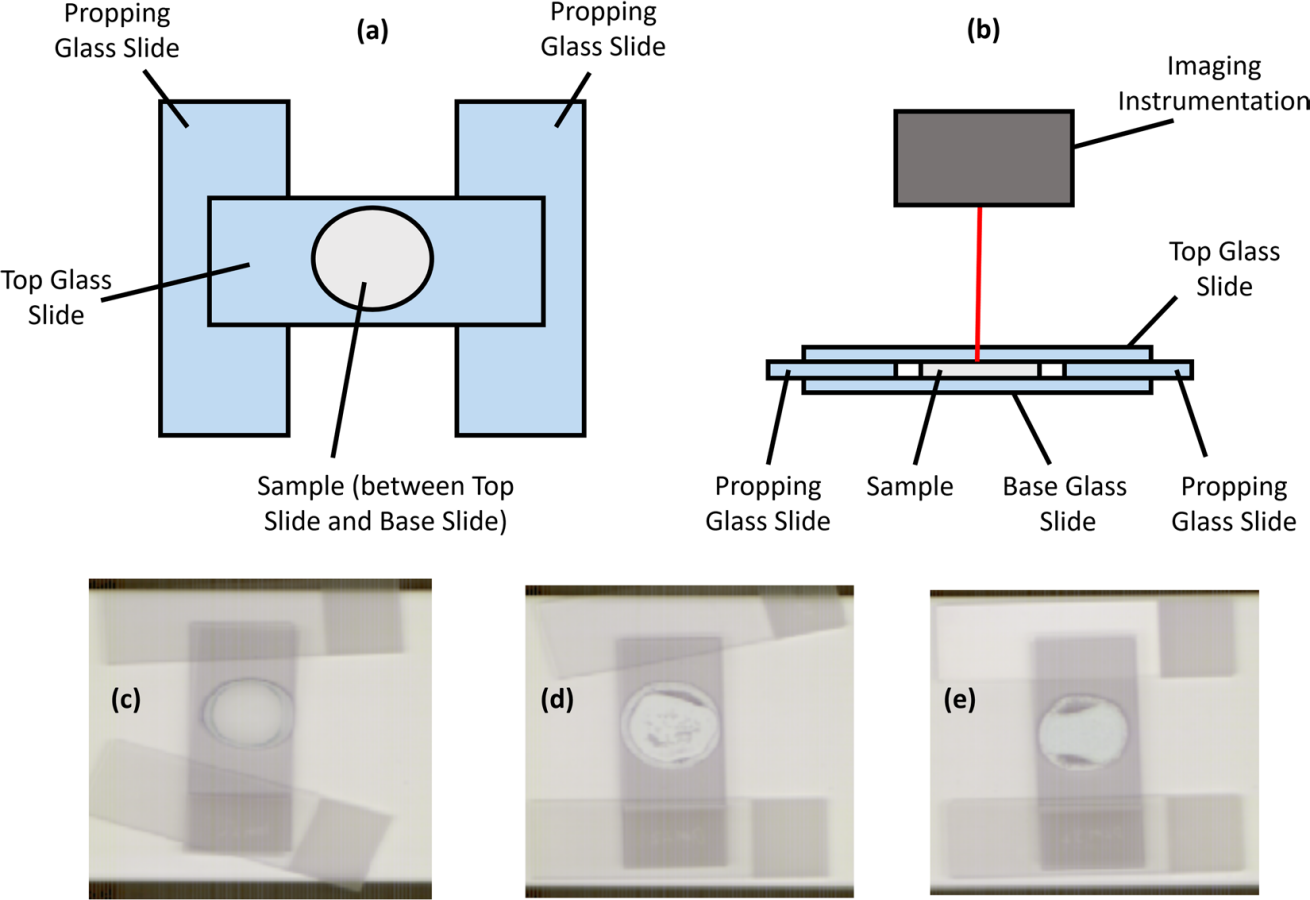
**(a)** Plan view of cement setting and imaging arrangement. **(b)** Elevation view of cement setting and imaging arrangement including imaging instrumentation. **(c–e)** Experimental setting and imaging arrangements (M0, M1 and M2 respectively).

Some of the paste of each cement type (approximately 0.5 mL) was pipetted onto the centre of a (base) glass slide. Two (propping) glass slides were placed at either end of the base glass slide and a final (top) glass slide was placed over the cement specimen, supported by the propping slides at either end of the base glass slide. Fig. 6 shows a schematic of the cement setting arrangement in plan and elevation views.

The cement specimens were left to set in air in this arrangement for 231 days before NIR image acquisition. Raman map acquisition was performed after 233 days. These time points were selected in order to ensure sample stability between Raman and NIR acquisition and to have a common setting period for all three cement types.

### Raman mapping

Raman chemical maps were acquired using an inVia Micro-Raman confocal spectroscopy system (Renishaw, Wotton-under-Edge, Gloucestershire, UK) with a 10 × 0.25 NA objective lens, 532 nm laser (500 mW, set to 50% power) and an 1800 line mm^−1^ grating. Laser spot size (D_xy_) was calculated as 1298 nm. Spectra were calibrated to a silicon shift at 520 cm^−1^. The detector used was a NIR enhanced Deep Depletion CCD array (1024 × 256 pixels) which was Peltier cooled to −70 °C. The spectral range was 2615.6–3916.6 cm^−1^ with a mean spectral resolution of 1.2830 cm^−1^ over 1015 measured spectral bands. As complete Raman imaging was not feasible, areas were mapped in the manner depicted in Supplementary Fig. 5, across the diameter of the cement in two orthogonal directions (traverse and longitudinal relative to the orientation of the glass slides). Spectra were acquired at steps of 100 μm in the mapping direction. Orthogonal to the mapping direction, lines were four or five pixels wide with a step size of 1 μm.

### NIR imaging

NIR chemical images were recorded using a line-mapping NIR hyperspectral imaging system (DV Optics, Padua, Italy), working in the range 11136–5814 cm^−1^ (880–1720 nm), where reflectance was measured every 7 nm (corresponding to a mean wavenumber resolution of 44.35 cm^−1^). Further details of this system can be found in Gowen *et al*.^27^. The pixel area was approximately 320 * 320 µm. Images were acquired in a push-broom manner with a stage velocity of 20 mm s^−1^. NIR images were acquired across an entire surface of each cement.

### Data Processing

All data analysis was carried out using Matlab (release R2017b, The MathWorks, Inc., Natick, MA, USA) incorporating functions from the Image Processing and Statistics toolboxes, as well as additional in-house functions which can be obtained from the authors upon request. Diagrams were arranged using Microsoft PowerPoint 2013.

#### Pre-Processing

NIR spectra were converted from reflectance to absorbance according to Eq. 1.

1 $${\rm{A}}={\mathrm{log}}_{10}(\frac{1}{R})$$

Conversion from reflectance to absorbance, where A is absorbance and R is reflectance.

Raman spectra were cut to the 3000 cm^−1^–3916.6 cm^−1^. Linear base-line correction (LBL) was performed on both NIR and Raman spectra, taking the first and last spectral values to define the linear base-line. This was followed by standard normal variate (SNV) normalisation. Cosmic rays (in Raman spectra) and spectral spikes (in NIR spectra) were corrected by replacement with the mean of neighbouring measured Raman shifts/wavenumbers. All NIR HCIs were masked to remove background pixels from consideration. NIR background pixels were identified using distinguishing absorbance value ratios (6845 cm^−1^–9542 cm^−1^) and masked by thresholding. The pre-processed spectra were used for all subsequent analysis.

### Data analysis

**Figure 7:**
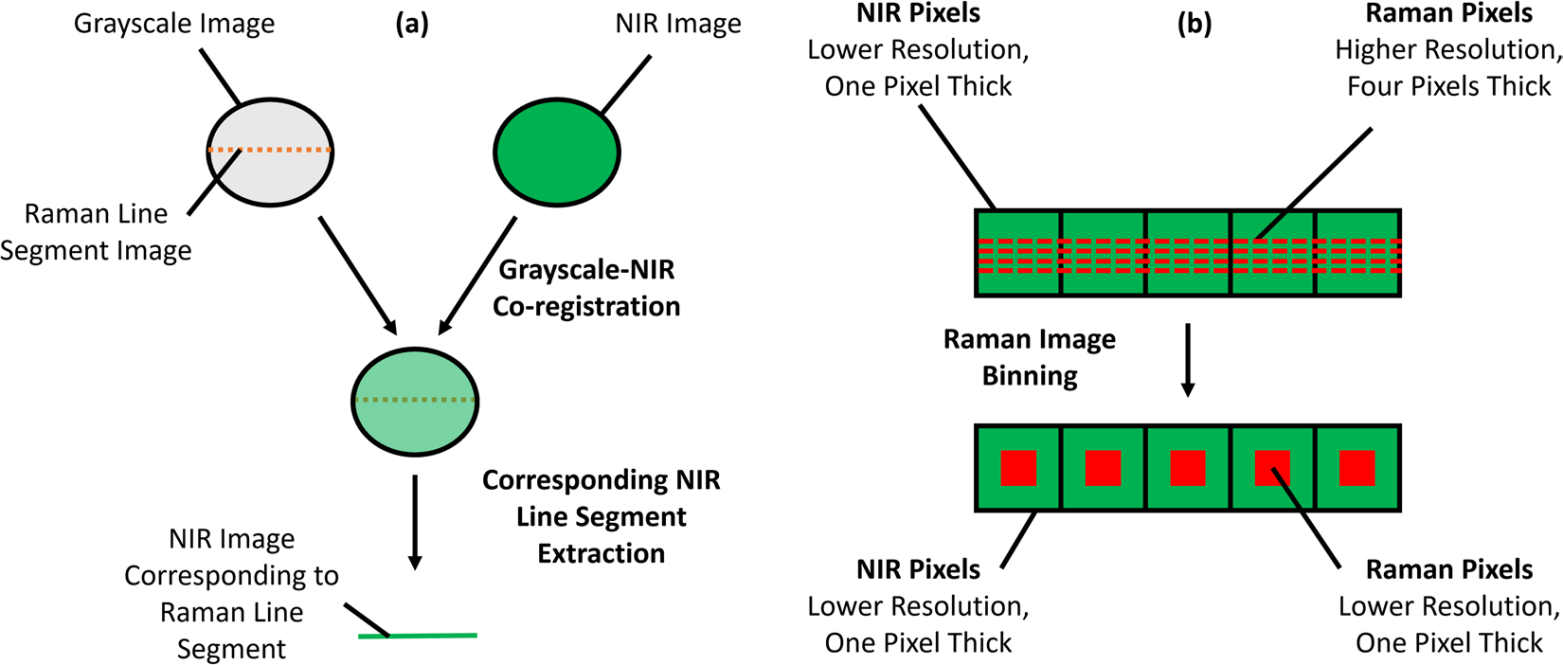
**(a)** Schematic representing the co-registration process and **(b)** schematic of Raman image pixel binning for the co-location of pixels between chemical imaging modalities.

As the Raman chemical maps were several pixels thick, mean spectra along the width dimension were calculated. After pre-processing, mean Raman and NIR spectra were calculated, and pixel spectra were inspected at different regions of the specimens. Intra-modality correlations were assessed by calculation of Pearson correlation coefficients. In order to conduct a comparative analysis between spectral features of Raman and NIR modalities, pixel co-registration of spatially co-located data was first necessary. As stated, Raman mapping was conducted through the sampling of line segments across diameters of the upper surface of the cement specimens (as depicted in Supplementary Fig. 5), while NIR imaging was conducted across the entire upper surface of the cement specimens. Co-registration was thus achieved by first limiting the NIR image spectra to those co-located with pixels of the Raman maps. All Raman maps were captured with spatial reference to grayscale images, which were acquired across the entire sample surface. These grayscale images were superimposed onto corresponding NIR images using identifying visual features as anchors. After grayscale-NIR image co-location, the relevant NIR image segment was recorded (Fig. 7).

The Raman laser spot size was smaller than the NIR pixel area. Further, the Raman pixel step-sizes were large relative to the pixel size at 100 μm, while the NIR pixels were spatially contiguous. For this reason, the Raman maps were binned such that they comprised the same number of pixels as the NIR line segment (Fig. 7(b)). As several Raman pixels co-located to each NIR pixel, the binning process calculated the mean value of Raman pixels which co-located to each NIR pixel, weighting spectra in instances of partial overlap between pixels.

After pixel registration, inter-modality Pearson correlation analysis was conducted. The Raman maps and NIR line segments were then independently concatenated into matrices and k-means clustering was performed in order to identify chemically different cement regions. For both Raman and NIR modalities, the optimal number of clusters was selected by plotting the percentage mean Euclidean distance of each data point to its cluster centroid relative to a single cluster system (mean of 10,000 iterations; 2 to 10 clusters assessed) in combination with the elbow method. Raman and NIR k-means cluster maps were generated and compared.

Finally, Non-Linear Iterative Partial Least Squares (NIPALS) regression^24,27–29^ was performed in order to assess the predictive properties of NIR spectra for Raman spectra. The model was both trained and calibrated using co-registered NIR and Raman spectra acquired in the transverse direction, and validated on co-registered NIR and Raman spectra acquired in the longitudinal direction. The number of latent variables selected was that which minimised the BM_r__RVM_r_ metric calculated on the calibration set (root mean square error of calibration (RMSEC) + absolute sum of differences between each regression vector element and its neighbouring element [i.e. a measure for vector “jaggedness”])^27^. The PLS regression vector was applied to the full NIR images and compared with the k-means results.

## Electronic supplementary material

Supplementary Information
