# Supplementary material for: Identification of Magnesium Oxychloride Cement Biomaterial Heterogeneity using Raman Chemical Mapping and NIR Hyperspectral Chemical Imaging
Source: Sci Rep. 2018 Aug 29;8:13034. doi: 10.1038/s41598-018-31379-5 (PMC6115415; doi:10.1038/s41598-018-31379-5)
Supplement: Supplementary file 1 — Supplementary Information [file 41598_2018_31379_MOESM1_ESM.docx]

**Identification of Magnesium Oxychloride Cement Biomaterial Heterogeneity using Raman Chemical Mapping and NIR Hyperspectral Chemical Imaging**

**Ronan M. Dorrepaal and Aoife A. Gowen**

UCD School of Biosystems and Food Engineering, University College Dublin, Ireland

*Corresponding author: Email: ronan.dorrepaal@ucdconnect.ie

Tel: +353 1 716 2601

**Supplementary Figures**


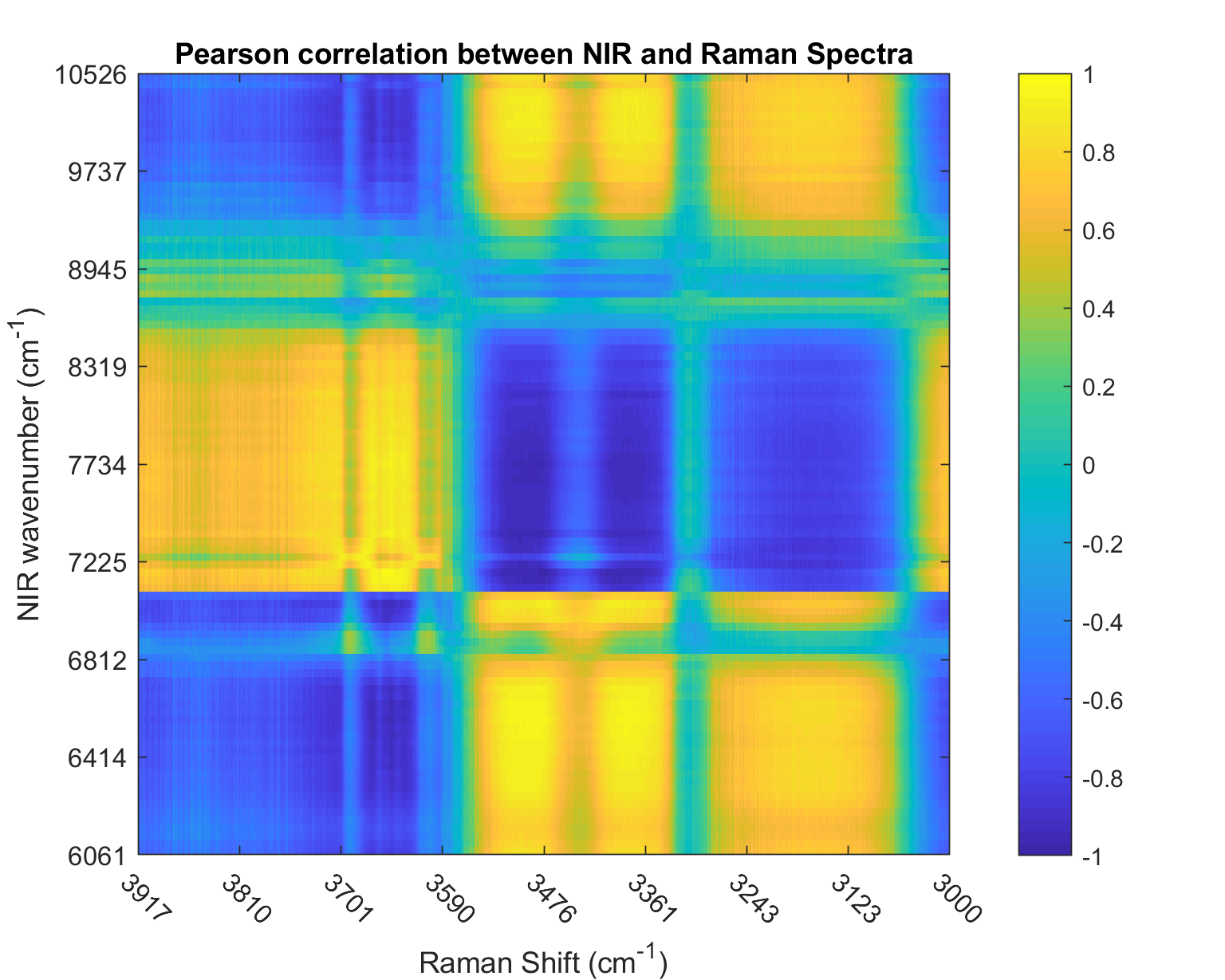


Supplementary Figure 1. Pearson correlation values between NIR wavenumbers in M0, M1 and M2 MOC with Raman shift.


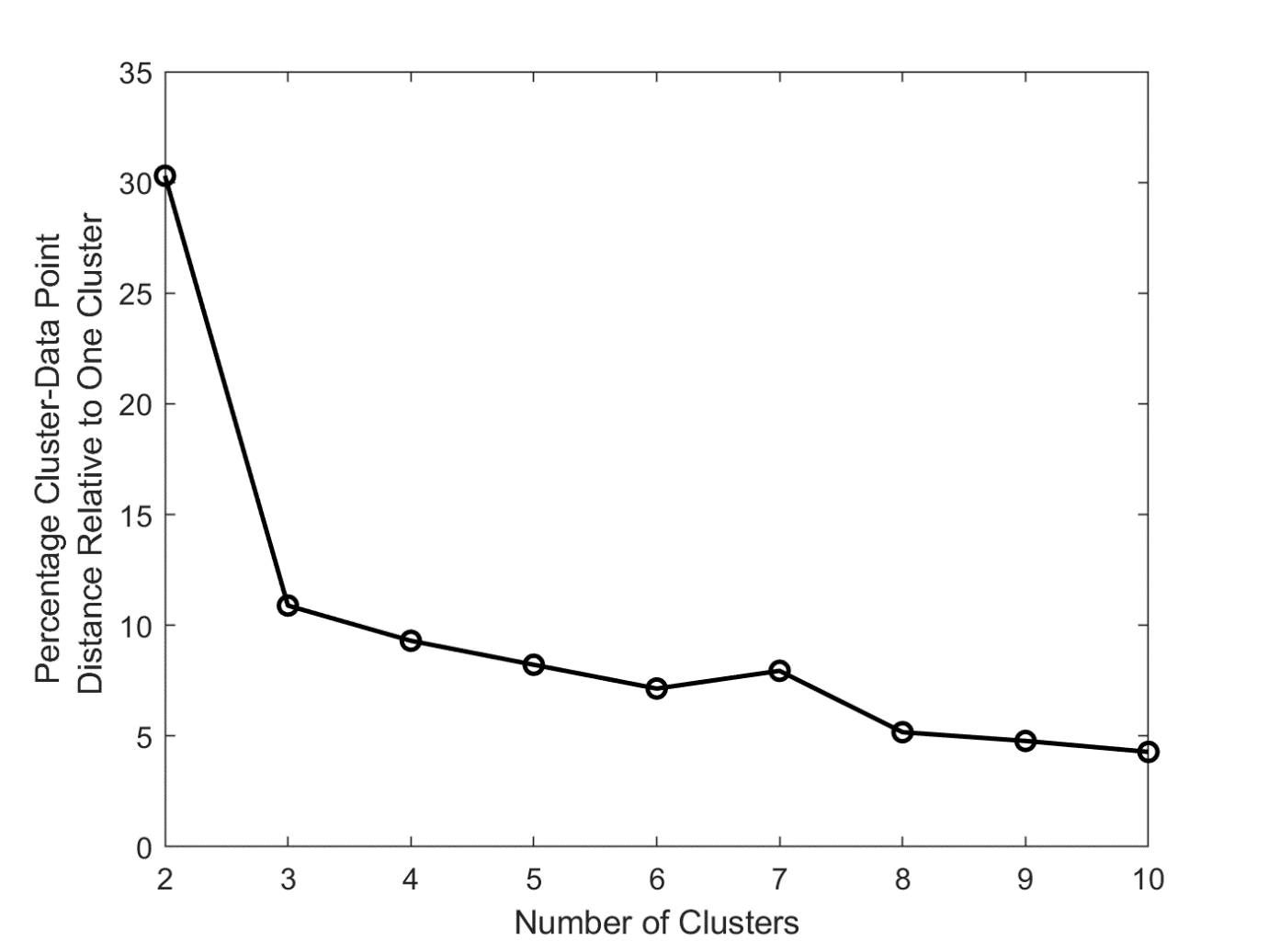


Supplementary Figure 2. Raman k-means cluster optimisation: Number of clusters vs cluster-data point distance relative to one cluster (%).


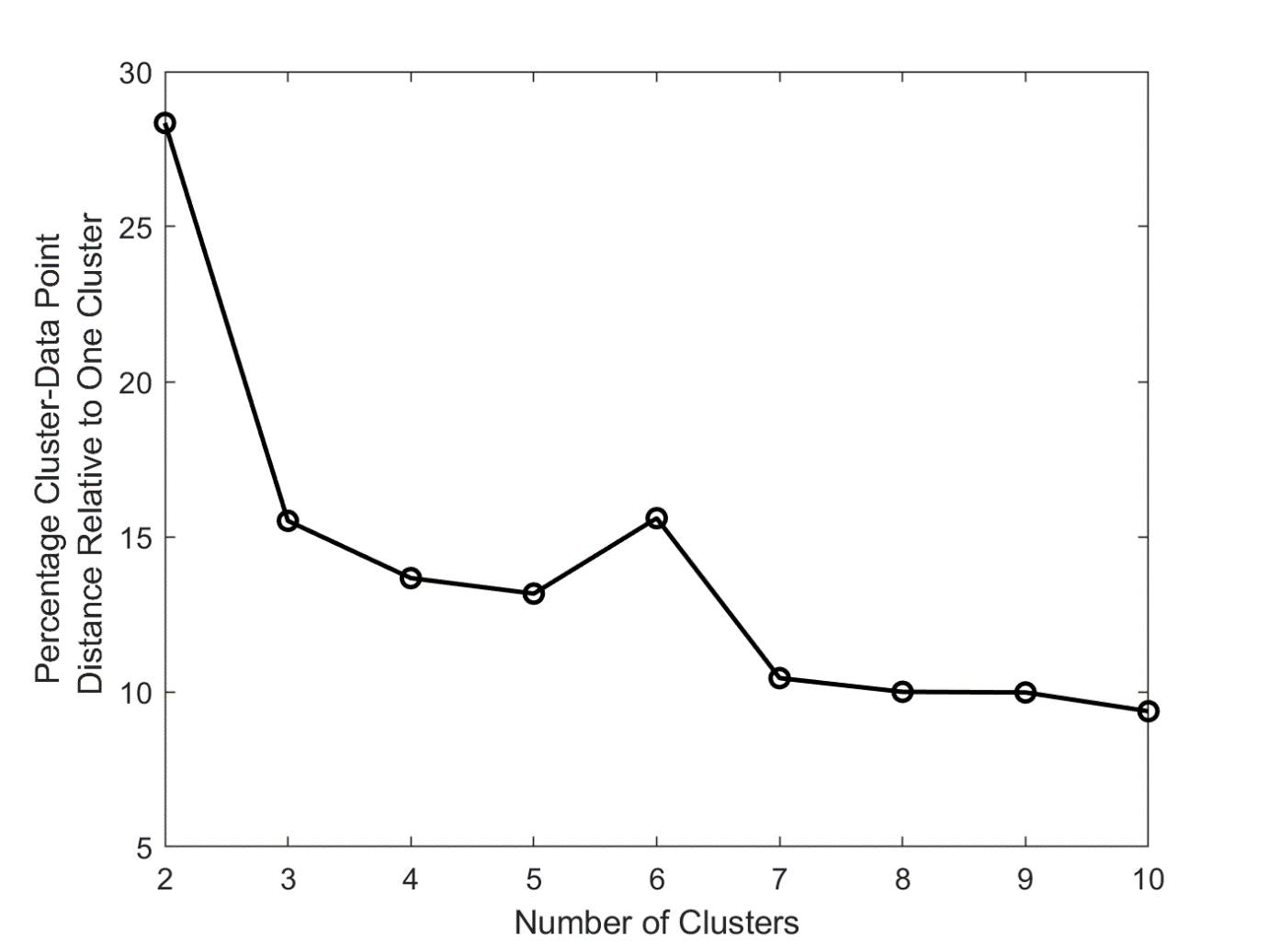


Supplementary Figure 3. NIR k-means cluster optimisation: Number of clusters vs cluster-data point distance relative to one cluster (%).


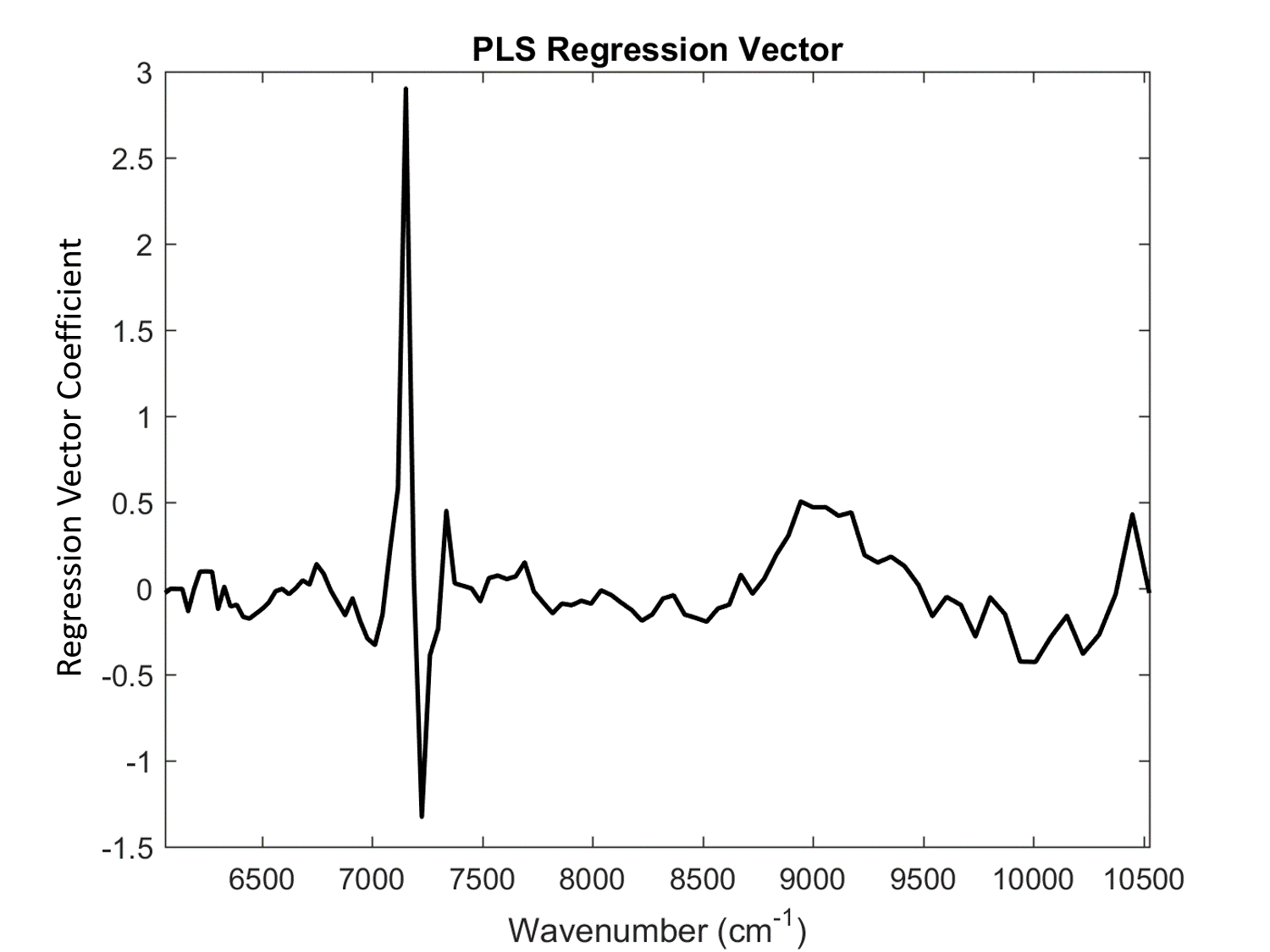


Supplementary Figure 4. PLS regression vector: For prediction of 3650 cm^-1^ Raman shift intensity from NIR spectrum.


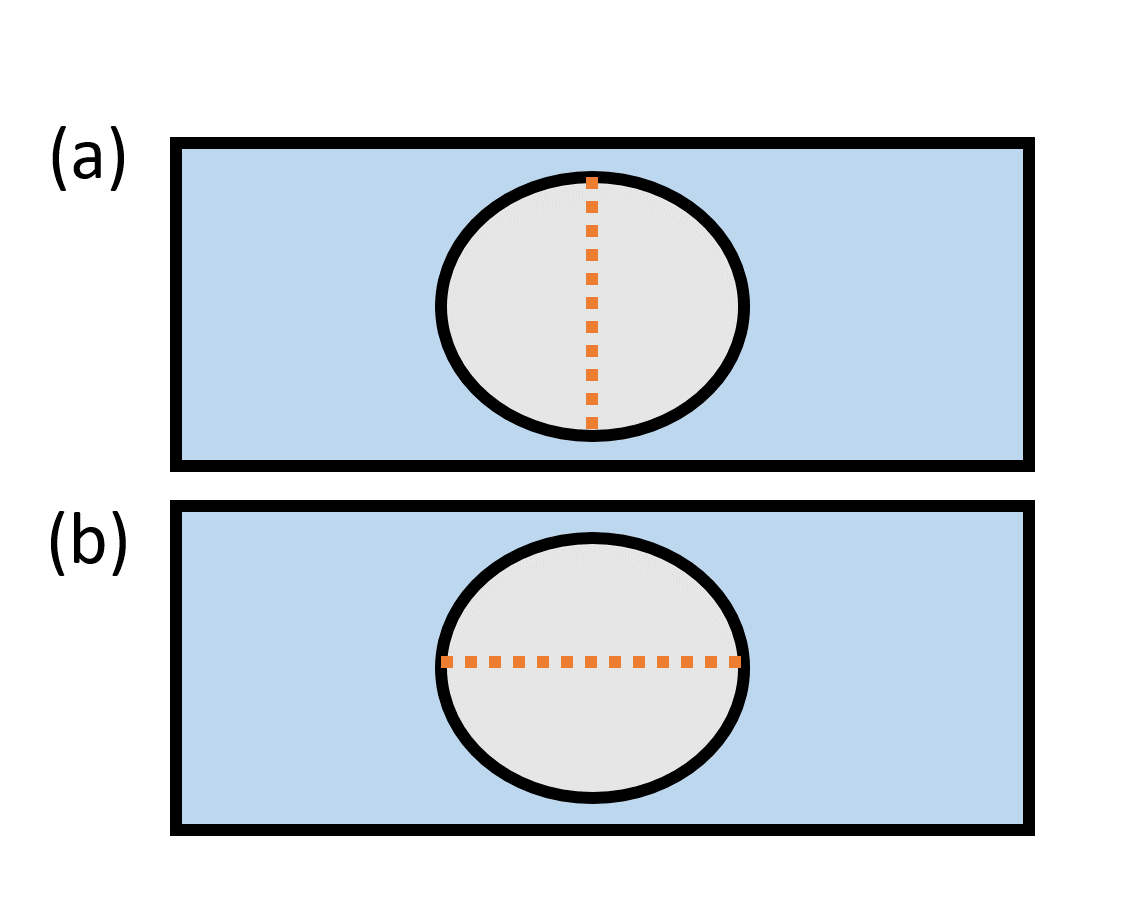


Supplementary Figure 5. (a) Cement section Raman imaged a transverse direction relative to the top glass slide, (b) cement section Raman imaged in the longitudinal direction relative to top the glass slide.

**Supplementary Tables**

| **Raman (cm^-1^)** |  |  |  |  |  |
| --- | --- | --- | --- | --- | --- |
| **M0** | **3608** | **3639** | **3650** | **3657** | **3691** |
| **3608** | - | -0.70 | -0.77 | -0.68 | 0.98 |
| **3639** | - | - | 0.67 | 0.95 | -0.72 |
| **3650** | - | - | - | 0.54 | -0.74 |
| **3657** | - | - | - | - | -0.69 |
| **3691** | - | - | - | - | - |
| **M1** | **3608** | **3639** | **3650** | **3657** | **3691** |
| **3608** | - | 0.88 | 0.96 | 0.77 | 0.99 |
| **3639** | - | - | 0.91 | 0.83 | 0.87 |
| **3650** | - | - | - | 0.79 | 0.95 |
| **3657** | - | - | - | - | 0.77 |
| **3691** | - | - | - | - | - |
| **M2** | **3608** | **3639** | **3650** | **3657** | **3691** |
| **3608** | - | 0.26 | 0.53 | 0.21 | 0.87 |
| **3639** | - | - | 0.73 | 0.95 | 0.23 |
| **3650** | - | - | - | 0.72 | 0.54 |
| **3657** | - | - | - | - | 0.19 |
| **3691** | - | - | - | - | - |
| **All** | **3608** | **3639** | **3650** | **3657** | **3691** |
| **3608** | - | 0.28 | 0.18 | 0.17 | 0.99 |
| **3639** | - | - | 0.92 | 0.98 | 0.24 |
| **3650** | - | - | - | 0.91 | 0.14 |
| **3657** | - | - | - | - | 0.13 |
| **3691** | - | - | - | - | - |

Supplementary Table 1. Pearson correlation values between peaks of interest in M0, M1 and M2 MOC cement Raman spectra (individual and combined).

| **NIR**  **(cm^-1^)** |  |  |  |  |
| --- | --- | --- | --- | --- |
| **M0** | **7336** | **7225** | **7189** | **7153** |
| **7336** | - | 0.66 | 0.89 | 0.94 |
| **7225** | - | - | 0.75 | 0.6 |
| **7189** | - | - | - | 0.92 |
| **7153** | - | - | - | - |
| **M1** | **7336** | **7225** | **7189** | **7153** |
| **7336** | - | 0.96 | 0.97 | 0.96 |
| **7225** | - | - | 0.99 | 0.99 |
| **7189** | - | - | - | 0.99 |
| **7153** | - | - | - | - |
| **M2** | **7336** | **7225** | **7189** | **7153** |
| **7336** | - | 0.64 | 0.63 | 0.57 |
| **7225** | - | - | 0.95 | 0.83 |
| **7189** | - | - | - | 0.89 |
| **7153** | - | - | - | - |
| **All** | **7336** | **7225** | **7189** | **7153** |
| **7336** | - | 0.84 | 0.91 | 0.94 |
| **7225** | - | - | 0.97 | 0.84 |
| **7189** | - | - | - | 0.93 |
| **7153** | - | - | - | - |

Supplementary Table 2. Pearson correlation values between peaks of interest in M0, M1 and M2 MOC cements NIR spectra (individual and combined).
